# Supplementary material for: Are carbon emissions trading and green financial instruments synergistic? -Comprehensive quantitative research based on content analysis
Source: PLoS One. 2024 Mar 7;19(3):e0298601. doi: 10.1371/journal.pone.0298601 (PMC10919601; doi:10.1371/journal.pone.0298601)
Supplement: S1 Data — (ZIP) [file pone.0298601.s001.zip › Data/地级市Z_F工作报告.zip!/╜¡╦╒/─╧╛⌐╩╨/└┤╫╘╣1⁄2╓┌║┼í╛┬φ┐╦╩2╛▌═°í┐_2014.docx]

各位代表：

　　现在，我代表市人民政府，向大会报告工作，请予审议，并请政协委员和其他列席人员提出意见。

　　一、2013年政府工作回顾

　　过去的一年，我们在市委的正确领导下，紧紧依靠全市人民，全面贯彻党中央国务院和省委省政府的决策部署，坚持“稳中求进、以进促稳”总基调，以提高经济增长质量效益为中心，积极应对各种压力挑战，扎实推进经济发展、科技创新、产业转型、城乡建设、环境保护、民生改善各项工作，较好完成十五届人大一次会议确定的目标任务。

　　过去的一年，我们实施了建国以来全市最大范围的行政区划调整，全域城市化、城乡一体化迈出新步伐；科学编制并推进实施苏南现代化建设示范区南京规划和三年计划，发展空间实现新拓展；坚持“办赛事、建城市、惠民生”，成功举办了第二届亚青会，城市功能和影响力得到新提升；认真开展群众路线教育实践活动，着力解决“四风”问题，作风建设取得新成效。

　　经济实现持续稳定增长。预计实现地区生产总值8000亿元，按可比价计算增长11%；实现公共财政预算收入831.3亿元，增长13.4%；完成全社会固定资产投资5250亿元，增长12%；完成社会消费品零售总额3500亿元，增长13.5%；城市居民人均可支配收入40000元，增长10%；农民人均纯收入16500元，增长12%；万元地区生产总值能耗下降8%；居民消费价格上涨2.7%，涨幅控制在省定标准以内。

　　产业转型升级步伐加快。科技创新成效明显，建成紫金科创特区200万平方米，在孵企业超过3000家；集聚“321”人才920名，新增世界500强和中国500强企业研发机构21家，集聚科技型企业1400家；专利申请总量超过4万件。产业结构持续优化，三次产业比例调整为2.3：43.3：54.4。软件、金融、广告创意、电子商务、服务外包等现代服务业保持良好发展态势，服务业增加值占地区生产总值比重提高1个百分点。工业加速转型升级，规模以上工业企业利税增长20%以上，战略性新兴产业增速超过规模工业增速一倍以上，产值首次超过石化、钢铁、建材三大传统产业。完成96个重点节能改造和循环经济项目，整治141家“三高两低”企业。

　　城市功能品质稳步提升。河西青奥村、海峡城等重点项目进展顺利，完成江北新区战略规划，仙林科技城协同创新集聚区建设取得突破。宁杭城际铁路开通运营，地铁3号线、4号线、10号线一期、宁高城际一期、宁天城际一期、宁和城际一期建设加快推进，溧马高速公路建成通车，122省道、江北大道、宁滁快速通道、宁高新通道等城乡大通道加快实施，纬三路过江隧道等建设进展顺利，城西干道、江东路快速化改造及南京南站枢纽快速环线工程基本完成。历史文化保护力度加大，新增国家级文保单位22个。江宁织造博物馆、老城南门东片区箍桶巷示范街、南京书画院、金陵美术馆、老城南记忆馆建成开放，明城墙玄武门至神策门段环境综合整治加快实施，六朝博物馆、科举博物馆、大报恩寺遗址公园、牛首山遗址公园等建设有序推进。实施城市治理条例，动迁拆违治乱整破年度任务顺利完成。智慧南京建设全面推进。成立南京都市圈发展联盟，完成都市圈区域规划。

　　城乡统筹发展成效明显。郊区发展实力不断增强，预计实现地区生产总值4120亿元，增长12.3%，占全市51.5%。先进制造业基地加快建设，液晶谷、未来网络谷、智能电网集聚区、桥林汽车产业园等一批重点项目加快推进。现代农业加速发展，启动11个市级现代农业园区建设，新增设施农业8万亩，新增农民合作社400家，生物农业产值达100亿元，家庭农场达1223家，建成国际慢城生态游等12条休闲农业精品线路，郊区旅游总收入达269亿元。推进45万亩土地综合整治，完成3510个村庄环境整治。启动“美丽乡村”五大片区规划建设。新增造林面积7.6万亩。高淳、溧水两区实现引江供水。完成长江干堤加固工程，以及30万亩农田水利建设。

　　改革开放取得积极进展。综合改革取得突破，第一阶段12项重点改革基本完成，202项市级行政权力下放到位。市区财政管理体制改革、公费医疗制度改革、科技体制改革稳步实施，市属国有企业改革、城市建设管理体制改革、转变土地利用方式试点、统筹城乡发展改革有序推进。对外开放进一步扩大，成功举办紫金山峰会等活动，利用外资质量效益同步提升，新增跨国公司地区总部等外资功能性机构12家。完成外贸出口总额320亿美元。南京综合保税区一期通过国家验收，金陵海关正式运作。实施“百优民企”培育计划，民营经济增加值占比提高1个百分点。

　　生态文明建设扎实推进。创成国家森林城市，通过国家生态市技术评审，编制出台生态文明建设规划。严格落实“五个最严”制度，狠抓节能降耗、污染减排，严控煤炭消耗总量和污染排放强度。实施蓝天清水工程，加强扬尘、工业废气、机动车尾气治理，强化渣土车整治，做好秸秆禁烧工作。扎实推进板桥地区、大厂地区及金陵石化周边工业污染综合整治。对雨污分流开展“回头看”，有序推进城市排水工程建设。推进20条黑臭河道整治。城镇污水日处理能力达200万吨。启动紫金山—玄武湖文化旅游示范区规划建设，滨江风光带、明外郭—秦淮新河风光带等建设加快。主城区垃圾分类全面推开，江南、江北两个环保产业园及其配套工程建设进展顺利。

　　民生社会事业全面发展。新增城镇就业20万人以上，城镇登记失业率控制在2.68%以下。高淳、溧水两区失业保险纳入市级统筹。企业退休人员人均养老金提高到2285元。新增养老床位5285张，建成1248个社区(村)居家养老服务中心。民生十件实事全部兑现，完成350万平方米城中村危旧房改造、135条街巷整治和45个农贸市场改造，新辟和调整优化公交线路61条，新建和改造农村公路283公里。启动县级公立医院改革，高中多样化办学和义务教育优质均衡发展领先全国。公共文化服务体系不断完善，文化产业增加值占比预计达到5.4%。建成保障房557万平方米，花岗、岱山、丁家庄、上坊等8万套保障房基本具备交付条件。完善了被征地农民老年生活保障和住房保障政策。法治南京、平安南京建设深入推进，城市公共安全应急机制和社会治安防控体系建设成效显著。食品药品安全工作得到加强。有效防控人感染H7N9禽流感。双拥优抚工作融合推进。计划生育、妇女儿童、爱国卫生、慈善、残疾人等各项事业取得新进步。

　　各位代表，过去的一年，我们经历了严峻考验，成绩来之不易。这是市委贯彻中央和省委决策，统揽全局、正确领导的结果，是全市广大干部群众团结奋进、持续拼搏的结果。在此，我代表市人民政府，向在各条战线付出辛勤劳动的全市人民，向给予大力支持、有效监督的人大代表、政协委员，以及各民主党派、工商联、无党派人士、人民团体和离退休老同志，向为南京改革发展稳定作出积极贡献的驻宁部队指战员、武警官兵、人民警察，向长期以来关心和支持南京发展的港澳台同胞、海外侨胞、国际友人，表示崇高的敬意和衷心的感谢!

　　在看到成绩的同时，我们更加清醒地认识到，经济社会发展还存在一些困难和问题。影响经济社会发展的体制机制问题尚未得到有效解决，综合改革工程仍需深入推进。优化经济结构的任务仍然艰巨，战略性新兴产业还处在培育发展阶段，服务业层次和水平还需提高。城市规划、建设较为粗放，管理的科学化、精细化水平亟待进一步提升，政府对重大建设项目决策的科学性、规范性、有序性有待加强。政府职能转变还不到位，对微观经济干预仍然过多，企业的市场主体地位没有真正确立，市场配置资源的决定性作用有待充分发挥。资源环境约束加剧，节能减排压力加大、任务繁重。同时，民生工作和社会治理还存在着一些薄弱环节，灰霾等环境污染、交通拥堵、就业结构性矛盾、住房保障供应、教育医疗和养老服务等方面，还存在不少亟待解决的问题。城乡居民增收难度加大，城乡区域之间基本公共服务还存在差距。

　　我们还清醒地认识到，政府自身建设仍存在着一些缺点和不足，少数政府部门和工作人员服务意识不强，对群众和基层反映的问题不能及时有效解决，推诿扯皮等现象时有发生。工作落实推进不到位，存在重形式、轻实效，重布置、轻执行等问题。形式主义、官僚主义、享乐主义、奢靡之风不同程度存在，极少数人甚至以权谋私、贪污腐败。对此，我们一定高度重视，切实采取有效措施，认真加以解决。

　　二、2014年主要目标任务

　　今年是全面深化改革、圆满完成“十二五”规划目标任务的重要之年，也是举办青奥会、建设新南京的决胜之年。做好今年政府工作，要全面贯彻落实党的十八大和十八届三中全会精神，紧紧围绕主题主线，以改革创新为统领，以提高质量效益为中心，以保障和改善民生为宗旨，深入实施苏南现代化建设示范区南京规划，统筹推进“稳增长、调结构、抓创新、促改革、惠民生”各项工作，全力打好确保“经济发展稳中向好、改革创新实质突破、青奥会精彩圆满”三场硬仗，努力开创科学发展新局面，为率先基本实现现代化、建设现代化国际性人文绿都奠定坚实基础。

　　各位代表，今年全市经济社会发展指标分为三大类：一是预期性指标，地区生产总值增长10.5%左右；公共财政预算收入同口径增长10.5%；全社会固定资产投资增长10.5%以上；社会消费品零售总额增长13%以上。二是调控性指标，服务业增加值占地区生产总值比重提高1个百分点以上；高新技术产业产值占规模以上工业产值比重提高1.5个百分点以上；文化产业增加值占地区生产总值比重提高0.3个百分点以上；全社会研发经费支出占地区生产总值比重提高0.3个百分点以上。三是约束性指标，城市居民人均可支配收入增长11%以上，农民人均纯收入增长12%以上；新增城镇就业中大学以上人员占比提高3个百分点以上；科技创业投资增长15%以上；居民消费价格涨幅控制在省定标准；商品住宅供地面积500公顷以上，保障性住房供地应保尽保；万元地区生产总值能耗下降7%以上，煤炭消耗总量下降3%左右，万元地区生产总值二氧化碳排放下降6%以上。这些发展指标，是市委市政府深入研判国内外形势和南京发展阶段性特征、广泛听取各方面意见基础上提出的，既保持合理的增长区间，又为深化改革、结构调整、可持续发展留出空间。提高约束性指标考核权重，引导全市上下切实把工作重心放到全面深化改革、加快发展方式转变上，实现实实在在、不留后遗症的增长。

　　做好今年工作，要牢牢把握稳中求进这个总基调。“稳”是基本要求，始终牢记发展使命，坚持科学发展的永恒主题，实现经济平稳较快增长，巩固扩大稳中向好势头；“进”是目标追求，推进产业发展高端化、资源配置市场化、城乡(区域)一体化、城市功能品质国际化，推动产业结构调整取得新进展、经济发展质量效益迈上新台阶、生态环境和民生改善取得新成效。

　　做好今年工作，要牢牢把握改革创新这个核心动力。坚持以改革统领全局，以创新驱动发展。在深化改革中发挥好市场配置资源的决定性作用，充分激发市场活力和内生增长动力；在科技创新中加快实现发展动力转换，充分激发企业、社会创新创业的潜能和激情，使创新驱动成为南京发展的强大引擎，增强城市可持续发展的能力。

　　做好今年工作，要牢牢把握民生幸福这个根本宗旨。坚持以人为中心的工作导向，始终站在群众的角度想问题、办事情，始终把群众满意作为最高标准，把群众的愿望和要求作为决策施政的根本依据，发展好维护好群众的发展权益、财富权益、环境权益，着力解决住房保障和供应、大气环境、水环境、城市交通、教育卫生养老等方面突出问题，让人民群众切身感受到变化和成效。

　　全面推进第二阶段综合改革工程是今年的重大任务。坚持把改革作为解决发展问题的总钥匙、总开关，贯穿于经济社会发展各个领域，着力通过改革更好地稳增长、调结构、抓创新、惠民生，为经济社会持续健康发展释放改革红利。按照市委统一部署，全面推进七大类48项改革任务，制定分类实施方案，明确时间表、路线图、责任人，确保在重要领域和关键环节取得实质性突破。深化经济体制改革，全面推进科技创新、人才管理、区域金融、市级功能区、财政体制、国土利用、对外开放、国资管理等领域改革，通过市场机制优化配置资源，加快构建“创新驱动、内生增长、绿色发展”的体制机制；深化政府管理体制改革，处理好政府与市场、政府与社会的关系以及政府层级间的关系，推进机构改革，切实简政放权，把能下放的权限都下放到区(园区)、基层，能交给市场和社会的公共服务都交给市场和社会，加快构建“两级政府、三级管理、权责一致”的政府治理体制；深化社会治理体制改革，建立健全法律法规，推进街道社区体制改革，培育发展社会组织，建立政府购买服务制度，完善社会诚信体系，全面深化教育、医疗卫生、社会保障等领域改革，加快构建“党委领导、政府负责、社会协同、公众参与、科技支撑、法治保障”的社会治理体系；深化城乡建设管理体制改革，推进城乡规划管理体制、城建投融资机制、综合管养机制、城市综合执法、农村产权制度、新型农业经营体系等改革，创新城乡(区域)一体化体制机制、都市圈协同发展机制，加快推进全域城市化、城乡一体化；推动生态文明建设，加快绿色发展、循环发展和低碳发展。

　　在全面深化改革同时，重点做好八个方面工作：

　　(一)强化创新驱动，实现经济平稳健康发展。突出科技创新驱动和消费、投资、出口协同拉动作用，全面激发市场主体活力，促进经济增长提质增效。

　　增强科技创新驱动力。深化科技人才体制改革，推进国家科技体制综合改革试点、国家创新型试点城市建设，完善科技创新政策，优化创新创业环境，充分释放科技创新潜力。突出企业创新主体地位，推动创新政策、创新资源、创新人才向企业集聚，培育100家高新技术企业和创新型企业。更多地运用市场和社会力量，高效配置创新资源。出台鼓励支持风险投资发展的政策措施，完善科技金融服务体系，健全产学研协同创新机制，支持企业与高校院所共建创新平台，建设好大学科技园。大力发展科技中介服务，实现科技服务业总收入340亿元。加快推进创新载体建设，高标准创建紫金人才管理改革试验区，制定紫金科技创业特别社区条例，新增孵化面积200万平方米，新增在孵企业1660家，新增战略性新兴产业创新中心13个。制定人才政策创新清单，加快集聚“321”人才，新增领军型科技创业人才780名以上。深入实施知识产权强市战略，打造知识产权服务业集聚发展试验区。

　　增强消费、投资、出口协同拉动力。着力培育消费新热点，以建设国家信息消费试点城市、电子商务示范城市、互联网试点城市、城市共同配送试点城市为契机，重点培育信息服务、网络购物、智能终端等新型业态，发展文化、教育、体育、养老、健康等消费热点。促进投资合理增长，引导投资方向、优化投资结构、改善投资管理服务，放开外资民资投资领域，集中力量推进200个事关全局、带动结构优化的重大项目。优化外贸结构，大力发展服务贸易和服务外包，用好江苏·南京外贸品牌商品展销会等平台，促进外贸平稳健康增长。支持有条件的企业走出去，用好“两个市场、两种资源”。

　　增强企业主体活力。加快国资国企市场化改革，推进股权多元化、运营专业化和国资从非主业中退出，增强国有企业活力和竞争力。加快工商登记制度改革，按照“非禁即入”准则，支持民营资本参与国有企业产权制度改革，鼓励发展民营资本控股的混合所有制企业，鼓励社会资本兴办高中等非义务教育阶段学校和医疗机构。发挥企业转型升级的主体作用，优选培育100家成长性好的战略性新兴企业，支持苏宁云商等千亿、百亿级龙头企业发展。强化中小微企业融资等服务保障，促进中小微企业快速成长。

　　(二)突出高端导向，加速产业转型升级。大力发展高端产业，着力打造高端优势，加快构建结构优化、附加值高、竞争力强的现代产业体系。

　　实施战略性新兴产业倍增计划。加快发展新型显示、北斗卫星导航、机器人、新能源汽车等六大类9个战略性新兴产业，实现主营业务收入5800亿元，增长23%。加快发展智能电网、未来网络产业，再新增两个千亿级的新兴产业。推动中电熊猫G108液晶面板等101个产业转型升级项目，培育一批百亿级创新型高新技术企业。实施新一轮“千企升级”，深化“两化”融合，改造提升石油化工、汽车制造等传统产业。争创全国质量强市示范城市，打造更多知名品牌。把化解产能过剩作为产业结构调整的重大任务，以钢铁、焦化、电镀、造纸、水泥、纺织等产业为重点，综合施策，扎实工作，坚决淘汰落后产能。

　　提速发展现代服务业。深入推进国家服务业综合改革试点城市建设，继续实施服务业倍增计划，大力发展互联网金融、现代物流、旅游会展、广告创意、电子商务、科技服务、商务服务等现代服务业。加快建设泛长三角区域金融中心，提升河西金融集聚区、新街口金融商务区能级，金融服务业增加值增长15%。发挥中国软件名城效应，重点抓好“一谷两园”软件载体建设，发展云计算、物联网、信息安全、移动互联网、大数据等新型业态，软件和信息服务业收入增长27%。加快发展现代物流业，推动长江-12.5米深水航道(南京段)疏浚，推进区港联动，建设南京长江航运物流中心，重点发展集装箱运输、公铁水联运、港口物流。加快建设高端商务载体，大力发展楼宇经济和总部经济。提升旅游业发展水平，规划建设17个旅游集聚区。高水平建设现代服务业集聚区，市级以上服务业集聚区经营收入、投资、就业人数增长均超过15%。

　　加快发展现代农业。构建新型农业经营体系，大力发展生物农业、休闲农业、高效设施农业，新增设施农业6万亩，重点建设白马农业谷等“一谷四园”，发挥园区示范带动效应。完善农村土地流转服务体系，新增农民合作社300家，农户参加经济合作组织比重达75%。支持种粮大户、家庭农场、专业合作社和农业龙头企业发展，农业龙头企业销售收入增长10%。继续实施农业“1115”工程，加快国土整治、农田水利建设、农业综合开发，建设高标准农田14万亩，农业机械化水平达84%。

　　推动开发区转型发展。全面对接上海自贸区，提升南京综合保税区运营水平。推进高新区创建国家创新型科技园区、空港枢纽经济区申报空港综合保税区、南京港申报汽车整车进口口岸，推动具备条件的省级开发区申报国家级开发区，加快海峡两岸科技工业园建设，争创海峡两岸产业协同和创新驱动发展试验区。积极争取中新生态科技岛成为省级改革开放试验区。创建跨境电子商务示范城市。加大招商引资力度，办好创洽会、金洽会、软博会等重大经贸活动，实行产业链招商，促进园区集中集聚集约发展。

　　(三)精心办好青奥盛会，提升城市国际化形象。坚持节俭、创新、绿色、低碳办会，确保青奥会圆满成功，呈现一场青春盛会、文化盛会和体育盛会。

　　加快推进青奥工程进度。按照时序节点，高质量完成地铁10号线一期、青奥村、体育雕塑、机场高速扩建等16项青奥重点工程，确保比赛场馆及配套工程按时按质交付。认真做好开闭幕式和火炬传递等活动的创意方案、演练彩排工作。

　　确保城市运行安全有序。按照办国际赛事的标准，精心组织好城市交通运行、食品安全、物流仓储、医疗服务、水电气保供、安全保卫、志愿服务等工作，营造全民参与青奥会、奉献青奥会、服务青奥会的浓厚氛围。大力实施竞赛场馆周边、城市主干道沿线、重点窗口地区的环境整治提升，切实做好城市亮化、绿化、美化工作。建立完善区域环境保护联防联控机制，确保青奥会期间城市空气质量达到国际赛事要求。

　　展示城市国际化形象。认真组织第4届青年奥林匹克文化节等活动，挖掘城市文化内涵，注重彰显人文理念，让“城市有品质、建筑有故事”。加大对外宣传和旅游促销力度，打造奥林匹克城市品牌，提升南京国际声誉，留下青奥宝贵遗产。大力弘扬讲文明、守秩序、团结友善、热情好客的良好风尚，以真诚的微笑、热忱的服务迎接四海宾朋。

　　(四)坚持建管并重，提升全域城市化、城乡(区域)一体化水平。切实转变城市建设发展方式，着力提升功能内涵，从主要偏重城区向城乡(区域)一体转变，从主要偏重建设向建管并重转变，从主要偏重规模向更加注重功能、民生、文化、生态建设转变。适当压缩城市建设规模，收拢五指、捏紧拳头，突出保青奥、保民生等重点。

　　加快全域城市化进程。实施苏南现代化建设示范区南京规划和三年计划，开展“两个试验、四个示范、两个试点”各项工作。启动江北新区建设，完成新区总体规划、控制性规划和重点地区城市设计，加快基础设施建设。继续推动河西新城、东山副城、仙林副城等建设发展，完善功能配套，提升城市集聚辐射力。重点推动高淳、溧水两区深层次融入主城。推进市级功能区体制改革，完善四大国家级开发区、仙林科技城、中国(南京)软件谷体制机制，建立河西新城、浦口新城、南部新城、麒麟科技创新园等功能区的市属区管体制，促进产城融合发展。深化南京都市圈城市合作，有序推进快速道路、轨道交通等区域基础设施建设，加快宁镇扬同城化，创建全国区域协调发展体制创新试验区。

　　聚焦城市建设重点。强化国家级综合交通枢纽功能，统筹推进禄口航空港、高铁枢纽港和江海联运港建设。完成城市快速道路改造提升工程，确保地铁3号线、宁高城际一期、宁天城际一期等4条轨道交通线路通车。实施雨花台区铁心桥—西善桥、栖霞区燕子矶、鼓楼区下关滨江、秦淮区绕城公路沿线、玄武区铁路以北等旧城成片更新改造，提升城市形象。加快建设智慧南京，实现主城区4G网络全覆盖。加强政府性债务管理，控制和化解建设性债务风险。

　　强化城市综合管理。创新城市管理模式，加快从城市管理向城市治理转变，从突击整治、运动式整治向制度化、长效化管理转变，从注重市容卫生管理向注重城市平面、立面、空间、水体、地下设施等综合管理转变。全面提升城市的规划管理、环境管理、交通管理、施工现场管理、安全生产管理水平。深化市容环境网格化、精细化、数字化管理，规范占道经营、车辆停放、路牌广告设置，加大城市道路、楼宇立面保洁清洗力度，大力整治油烟、噪音扰民，提升城市品位和城市形象。加强工地和作业现场管理，下大力气控制扬尘污染。加大交通秩序治理，突出重点，创新突破，条块联动，社会互动，有效缓解中心城区交通压力。完善市政设施，推进管养分离，加强日常管护，塑造整洁优美的城市环境。

　　加快现代化新农村建设。推进城市基础设施和公共服务向农村延伸，加快城乡社会保障、交通水务、户籍制度一体化。深化转变土地利用方式创新试点、城镇低效用地再开发试点，继续开展农村土地承包经营权确权登记，探索农村宅基地有偿退出和利用办法，探索推进农民住房财产抵押、担保、转让。以江宁横溪、浦口桥林等10个统筹城乡发展试点镇街和新市镇建设为核心，加快推进新型城镇化。完成“美丽乡村”五大片区300平方公里示范区建设。建成干线公路150公里以上，建成宁高新通道主线，完成500公里农村公路提档升级。做好防汛抗旱工作，完成滁河三期干堤提升工程，基本完成水阳江高淳段整治，实施秦淮东河、石臼湖及固城湖堤防加固和长江新济州河段建设。

　　(五)加快生态文明建设，共建美丽南京新家园。以对人民群众认真负责的态度，下更大的决心、花更大的力气、采取更有力的措施，促进生态环境改善取得实实在在成效。

　　优化城市生态空间布局。坚持“多心开敞、轴向组团、拥江发展”的城市空间结构，科学有序地实施空间开发，构建可持续发展的生产空间、生活空间和生态空间。落实主体功能区战略，按照城中、东南、西南、江北和南部五大片区功能定位，引导各片区优化发展、保护生态。严守生态红线，严格管制禁止开发和限制开发区域，重点保护风景名胜区、森林公园、湿地公园等重要区域。

　　实施环境综合治理。加大节能减排力度，削减煤炭消耗总量，全面禁止新建燃用煤炭、重油、焦油等重污染燃料设施，全面推进燃煤锅炉清洁能源改造。深入整治“三高两低”和“两高一资”企业，完成扬子石化、金陵石化、南钢等6个重点脱硫、脱硝减排项目。加大空气污染治理力度，以灰霾治理为重点，铁腕治理工业废气、机动车尾气、施工扬尘，切实做好秸杆禁烧和综合利用工作。加大水环境治理力度，推进污水处理设施及收集管网建设，落实河长制，尽快消除城区河道黑臭现象。加大城乡环境综合治理力度，推进城中村、危旧房改造，改变环境脏乱差现象。推动垃圾分类处理，建成江南、江北环保产业园垃圾焚烧发电项目，生活垃圾分类收集覆盖率达到70%。

　　加强生态保护和建设。全力创建国家生态市和生态园林城市。精心打造紫金山—玄武湖文化旅游示范区，继续推进滨江风光带、明外郭—秦淮新河风光带等建设。建成绿道100公里，创建50条林荫道，新增20个游园绿地，完善城市慢行系统、自行车道网络。推进河西绿色生态示范城区建设，建成300万平方米绿色建筑。完成绿化造林面积6万亩，林木覆盖率提高到28.9%。开展生态修复，关闭治理露采矿山宕口40个。弘扬生态文化，提高环境保护的公众参与度。

　　加强生态文明制度建设。完善重点生态功能区生态补偿、废弃资源处理补偿机制，制定实施主要污染物排放权有偿使用和排污权交易办法，组建排污权交易中心。实行严格的环保准入制度，加大环保执法力度，严肃查处环境违法行为。

　　(六)推进文化强市建设，增强城市软实力。激发文化创造活力，增强文化凝聚力，充分彰显南京人文古都魅力。

　　保护城市历史文化。严格实施历史文化名城保护规划和紫线管理，全面启动苏南现代化建设示范区文化遗产保护试点工作，做好文化遗产保护和传承。建立市级城墙保护中心，整治明城墙周边环境，实现明城墙统一管理、对外开放，推进明清城墙和海上丝绸之路南京遗迹申遗工作。建成六朝博物馆、科举博物馆一期、南京直立猿人化石遗址博物馆等，完成大报恩寺遗址公园一期和牛首山遗址公园一期工程，实施百子亭风貌区等重要近现代建筑保护整理。

　　加强公共文化建设。弘扬社会主义核心价值观，深入开展文明城市创建。实施文化惠民“百千万”行动，丰富提升群众文化生活。完善文化基础设施和公共文化服务网络，创建省级公共文化服务体系示范区和国家示范项目，推进市广播发射台及玄武、六合、溧水等区级文化馆和图书馆新建扩建，推动“示范文化站”建设。组建南京市博物馆总馆，推进公共图书馆数字化和总分馆制建设，举办2014年“国际博物馆日”、“中国文化遗产日”和世界历史文化名城博览会。

　　发展壮大文化产业。深化市属国有文化集团改革，培育壮大骨干文化企业。促进文化与科技、金融、旅游等深度融合发展，加快数字影音娱乐、现代创意设计、新兴网络传媒、智慧旅游休闲等文化产业发展，推进南京创意设计中心一期、秦淮特色文化产业园、长江路历史文化休闲街区等文创集聚区建设，启动栖霞山文化休闲旅游度假区规划建设，文化产业增加值占地区生产总值比重超过5.7%。

　　(七)创新社会治理，促进社会和谐稳定。坚持系统治理、依法治理、综合治理、源头治理，提高社会治理水平，促进社会长治久安。

　　加强社会治理基础工作。推进社会管理重心下移，深化街道社区体制改革，开展社区“去行政化”改革试点，减轻社区负担，建设智慧社区、和谐社区。加强社会组织的培育和管理，大力发展商会类、科技类、公益慈善类、城乡社区服务类等社会组织。加强城市志愿服务站点建设，新增300支应急救援志愿服务队。加快建立信用基础数据库和服务平台，促进社会诚信体系建设。

　　加大社会矛盾调处力度。建立畅通的诉求表达、矛盾调处、权益保障机制，充分利用政务微博、市长信箱、网上论坛等网络平台，不断拓宽社会公众民意表达、参政议政渠道。开展社会矛盾大排除、大调解，有效化解社会矛盾。全面推进和谐信访建设，落实信访事项首办首问负责制，着力化解信访积案。加强和完善网络信息管理，营造良好的网络环境。

　　切实保障城市公共安全。继续推进平安南京、法治南京建设，加强社会治安综合治理，严密防范、依法打击各类违法犯罪活动。严格落实安全生产责任制，强化安全生产隐患排查治理，坚决遏制重特大安全事故发生。完善危机预防和管理体系，提高对自然灾害、事故灾难、公共卫生和社会安全事件的应急处置能力。加强食品药品安全监管，主要食品和基本药物检测覆盖率分别达80%、100%。

　　(八)保障和改善民生，不断增进民生福祉。立足当前办实事、着眼根本建制度，着力构建普惠、均衡、优质的公共服务体系。

　　促进创业就业富民增收。统筹抓好农村劳动力转移、城镇就业困难人员就业，把吸纳高校毕业生就业放到更加突出的位置，新增城镇就业16万人，实现再就业6.5万人，支持更多的大学生创业，城镇登记失业率控制在4%以内。千方百计增加城乡居民收入，实施“就业提升、创业富民、财富增值、民生普惠”四项工程，拓展“就业、创业、投资、社保和帮扶”五大增收渠道。组织开展各类职业培训30万人次。

　　提高城乡社会保障水平。继续完善社会保障体系，社会保险五项险种参保人员净增30万人次，确保城乡基本养老保险、基本医疗保险、失业保险覆盖率达到98%以上。继续做好被征地人员的社会保障工作。全面推进医保省内异地联网即时结算，完善市、区职工医保同城联网结算。健全社会救助体系，加快残疾人康复托养机构建设，发展社会公益慈善事业。

　　办好人民满意的教育。改革小升初、高中学业水平考试和中考办法。调整幼儿助学券制度，推行学前一年基本免费教育，加快发展学前教育。调整优化教育空间布局，推进义务教育优质均衡发展，优质教育资源覆盖率达到80%。整合发展职业教育，推动市民终身教育，加快建设学习型城市。做好外来务工人员子女就学工作。

　　提高群众健康水平。普及卫生防病知识，强化市民健康意识。规划建设生命科技产业和健康服务名城，加快建设河西新城、南部新城等片区医疗中心和市公共卫生医疗中心。全面推行公立医院改革，推进基层医疗机构标准化建设。提高新农合筹资标准。有序启动“单独二孩”生育政策。广泛开展群众性体育活动，城市社区“10分钟体育健身圈”建设覆盖率达到100%。

　　大力发展养老事业。发展社区居家养老服务中心，新增养老床位3900张以上，每千名老人床位达到37张。重点发展医护型养老机构，加快建设市社会福利综合服务中心和青龙山老年康复中心。

　　做好城市必需品保供。完善粮食储备体系，确保全市粮食安全。推进永久性标准化蔬菜基地建设，丰富城乡居民“菜篮子”。实行价格补贴联动机制，降低物价上涨对低收入困难家庭基本生活的影响。加强供水、供气、供电等公用设施建设。改造农贸市场20个。

　　发展各项社会事业。继续做好民族宗教工作。深化“双拥共建”活动，促进军民融合发展。扎实做好援疆援藏工作，推进宁淮挂钩合作。认真做好保密、参事、国家安全、消防、人防、侨务、外事、档案、史志、气象、新闻出版、工青妇、社会科学等工作。

　　各位代表，人民群众的期盼，就是政府工作的方向。今年我们将重点办好保障和改善民生十件实事：一是在公交优先方面，新购更新公交车1000辆，新辟优化调整公交线路20条以上，建设公交专用道20公里，新建改造公交场站15个，出台公交换乘优惠措施，主城区居民公共交通出行分担率达到46%。新增停车泊位6万个。在主城以及河西、浦口等地增加公共自行车网点。二是在环境改善方面，淘汰落后产能企业30家，淘汰黄标车3.5万辆，整治南十里长沟。全面实现在运渣土车密闭化改造，工地达到扬尘污染控制标准。三是在保障性住房方面，续建各类保障性住房800万平方米，新开工保障房300万平方米，竣工300万平方米。四是在养老服务方面，建成市居家养老服务综合中心、91个3A级社区居家养老服务中心，新增医护型居家养老服务中心90个。五是在教育惠民方面，新改扩建幼儿园40所，新改建中小学校舍30万平方米。六是在医疗保障方面，城镇职工医保和居民医保制度规定范围内住院费用报销比例分别提高到84%和74%。七是在关爱残疾人方面，实现0—6岁残疾儿童抢救性康复全覆盖，新增残疾人就业1000人，为12万名残疾人购买免费乘车意外伤害保险。八是在提高社会福利方面，把低保对象医疗救助比例提高到60%，封顶线提高到3万元。农村五保户集中、分散供养标准分别达到当地农村低保标准的1.6倍和1.3倍。九是在旧城改造方面，完成城中村改造100万平方米、危旧房改造40万平方米，实施二次供水设施改造。十是在低收入农户增收方面，确保3.6万农村低收入人口，达到年人均纯收入6500元的脱贫标准。

　　三、加强政府改革建设

　　切实转变政府职能，推进简政放权，加强管理创新，提高治理能力，切实做好政府行政权力的减法，换取市场活力、社会活力的加法，提升政府的公信力、执行力、运作力，以及拒腐防变能力，以实实在在的业绩，赢得人民群众的信任。

　　增强政府公信力。推进政府机构改革，优化政府职能，减少对微观事务管理，减少政府对资源的直接配置，切实把该放的权放开放到位，把该管的事管住管得好。深化行政审批制度改革，建立行政审批目录清单，最大限度减少审批，努力营造统一开放、竞争有序的市场环境。坚持依法行政，运用法治思维、法治方式、法治手段管理经济社会事务，严格按照法定权限和程序行使权力、履行职责。坚持科学决策、民主决策、依法决策，加强调查研究，建立完善政府议事决策规则，完善重大行政决策咨询论证和公众征询制度，发挥好智库作用，积极推动网络问政。认真办理人大、政协建议提案，自觉接受人大及其常委会的法律监督与工作监督，自觉接受政协的民主监督，以及社会公众和舆论监督。开展好政府机关“公众开放日”活动。

　　强化政府执行力。巩固群众路线教育实践成果，坚决反对“四风”，深入基层，深入一线，推动作风建设常态化、长效化。牢固树立刚性化、绩效化、精细化的执行理念，深入推行“一线工作法”、“条线工作法”、“项目工作法”，确保重大决策、重大项目、重点工作不折不扣落到实处。严格实行导向鲜明的考核体系，强化目标刚性约束，发挥目标考核的激励奖惩功能，建立大督查机制，真正以目标引领行动、用督查推动落实。做好政策项目对接，争取国家和省更多的政策资金支持。切实改进文风会风，精简会议和文件，严格控制节庆论坛展会活动，把更多的精力用在抓落实上。

　　提升政府运作力。更多运用市场化、社会化、信息化方式提高行政效能。扩大政府向社会购买公共服务，制定政府购买公共服务指导目录，凡属事务管理性服务，原则上都引入竞争机制，通过合同委托等方式向社会购买。精心组织第三次全国经济普查，提供科学准确的统计数据。推进公共资源市场化配置，制定公共资源交易目录，推动经营性户外广告、行政及事业单位资产等市场化运作。深化财政体制改革，完善财政资金配置机制，提供更多更好更优的公共产品和公共服务。加快电子政务建设，实现信息共享共用。优化政务服务流程，充分发挥政务服务中心和“12345”政务热线作用，打造综合高效优质的政务服务平台。

　　提高拒腐防变能力。严格执行中央八项规定、省委十项规定，切实遵守市委“十七个一律”和“十一个一律”，建立反对“四风”长效机制。落实好“一岗双责”，完善权力制衡机制，真正把权力关进制度的笼子。认真执行廉政准则，强化效能监察和审计监督，严肃查处重点领域、关键环节腐败案件，对腐败问题“零容忍”。严控“三公”经费支出，严控楼堂馆所建设，切实把艰苦奋斗、厉行节约的要求，落实到政府运行各个环节。深入推进阳光政务，加快政府信息公开，除涉密部门外，市级政府组成部门预算全部公开。

　　各位代表，新的征程已经开启，新的目标催人奋进。让我们紧密团结在以习近平同志为总书记的党中央周围，在省委省政府和市委的领导下，解放思想、开拓创新，团结协作、真抓实干，为建设现代化国际性人文绿都而不懈奋斗!
